# The role of skin biopsy in differentiating idiopathic Parkinson’s disease from other types of parkinsonism

**DOI:** 10.1007/s00415-015-7974-2

**Published:** 2015-11-17

**Authors:** Ray Wynford-Thomas, Neil P. Robertson

**Affiliations:** Department of Neurology, Institute of Psychological Medicine and Clinical Neuroscience, Cardiff University, Cardiff, UK

## Introduction

Parkinson’s disease is one of the most common neurodegenerative disorders with a worldwide distribution. It has variable and complex phenotype with motor and non-motor manifestations, causes considerable psychosocial morbidity and consumes significant health care resources. Changing population demographics, and in particular, an ageing population in western societies is likely to result in gradually increasing prevalence. Despite significant advances in the understanding of disease pathology, the diagnosis of idiopathic Parkinson’s disease (IPD) remains largely clinical, with discriminatory investigations such as Ioflupane (^123^I)-labelled single-photon emission computed tomography (DaTSCAN) being used only in the minority of patients. However, postmortem studies have historically demonstrated the fallibility of relying on clinical phenotype and in differentiating IPD from ‘Parkinson’s plus’ conditions such as multiple system atrophy (MSA). An in vivo accessible peripheral biomarker for IPD and MSA would, therefore, be of great value to direct therapy and more accurate patient counselling.

The three papers discussed below assess the value of one of the most promising of the available candidate biomarkers in this context; phosphorylated alpha-synuclein (p-α-Syn) which has recently been identified within dermal nerve fibres in IPD. In the first paper, the authors examine the utility of p-α-Syn as a biomarker for diagnosis of IPD, the second looks at p-α-Syn as a biomarker for MSA and the third at using p-α-Syn to differentiate IPD from MSA.

## Skin nerve alpha-synuclein deposits: a biomarker for idiopathic Parkinson’s disease

This paper focuses on two hypotheses: whether p-α-Syn deposits in skin nerve fibres represent a useful biomarker for IPD and whether there is a link between these deposits and the underlying pathogenesis of peripheral neuropathy associated with IPD. The latter is based on experimental data showing that an accumulation of p-α-Syn deposits leads to neuron death. Twenty-one patients with well-characterised IPD (20 of whom had the diagnosis supported by cardiac uptake of [^123^I]-metaiodobenzylguanidine), 20 patients with parkinsonism (PAR) of different pathogenesis assumed not to have p-α-Syn deposits (vascular parkinsonism 10, tauopathies 6, parkin mutations 4) and 30 age-matched healthy controls were recruited for the study.

Three-millimetre punch biopsies of skin nerve fibres were taken at a proximal site (cervical C8 paravertebral area) and distal sites (thigh and distal leg). A second biopsy was also taken to assess variability of p-α-Syn expression from all areas in IPD patients, but only at the cervical area in the two other groups. No p-α-Syn deposits were identified in skin samples taken from the control or PAR groups. In contrast, when both skin samples were considered from the cervical area in the IPD group all patients were positive for p-α-Syn deposits. However, samples from thigh and distal leg sites in IPD patients were positive in only 52 and 24 %, respectively.

In the IPD group, p-α-Syn deposits showed a significant indirect correlation with leg epidermal innervation. Deposits were not correlated with age, disease duration, or Unified Parkinson’s Disease Rating Scale (UPDRS) score. The authors conclude that p-α-Syn deposits in proximal skin nerve fibres are a sensitive biomarker for the diagnosis of IPD and can help differentiate between other forms of parkinsonism. As a result of variable local expression, the highest sensitivity (100 %) was achieved by analysing two cervical skin samples. The authors also concluded that neuritic synuclein inclusions were correlated with a small fibre neuropathy, suggesting a possible direct role of p-α-Syn in peripheral nerve damage.

*Comment.* The use of skin biopsy in the diagnosis of IPD is an intriguing prospect. It is easily performed and minimally invasive with few adverse effects. This study provides some compelling evidence for use of skin biopsy in the diagnosis of IPD, although clearly larger studies would be of value in validating its use in clinical practice. The mean disease duration in the IPD group was 13 years, but it may be interesting to explore this biomarker in early or preclinical disease which may have significant implications for guiding earlier therapeutic interventions. However, the insights provided into pathophysiology remain limited since p-α-Syn deposits did not appear to correlate with disease duration or sub-phenotype.

Donadio V et al. (2014) Neurology 82:1362–1369.

## Distinctive distribution of phospho-alpha-synuclein in dermal nerves in multiple system atrophy

MSA also remains a largely clinical diagnosis with support from autonomic function tests and neuroradiology. Pathologically MSA is characterised by detection of α-Syn deposits in oligodendrocytes and neurons. In this study, Doppler et al. explore whether p-α-Syn deposits in dermal nerve fibres can be used as a biomarker in MSA and whether this could differentiate MSA from tauopathies and IPD. Twelve patients with probable MSA according to the Gilman criteria (6 where MSA was supported by neuroimaging and a further 6 without MRI support), 15 patients with presumed tauopathies (based on the criteria of Armstrong et al.), 30 patients with IPD (based on the UK Brain Bank criteria) and 39 healthy controls were examined.

Five-millimetre punch skin biopsies were taken from the back (Th12) and distal and proximal leg. Serial sections were undertaken to determine sensitivity and one in ten sections were double stained. p-α-Syn within dermal nerve fibres was detected in 8/12 patients with a clinical diagnosis of MSA. No p-α-Syn immunoreactive nerve fibres were identified in patients with presumed tauopathies or normal controls (p < 0.05). The sensitivity of skin biopsies stained positively for p-α-Syn in MSA was 67 % and specificity for a diagnosis of MSA versus tauopathies/controls was 100 %. In the IPD group, p-α-Syn was detected in 20 out of 30 patients, resulting in a sensitivity of 67 %. Double-staining serial sections of all p-α-Syn-negative patients identified one further patient with MSA and two more patients with IPD, increasing sensitivity to 75 and 73 %, respectively.

In MSA patients, p-α-Syn was predominantly deposited in somatosensory fibres of the subepidermal plexus, in contrast to IPD where autonomic fibres were predominantly affected. In the IPD group, proximal biopsy sites showed the highest detection rate, raising the possibility of antidromic spreading of p-α-Syn from ganglia to sensory nerve findings. The authors conclude that detection of p-α-Syn in cutaneous nerve fibres may support the diagnosis of a ‘synucleinopathy’ in contrast to tauopathies. They also suggest that p-α-Syn in somatosensory fibres is associated with MSA-parkinsonism, whereas autonomic fibres are involved in IPD.

*Comment.* This study advocates the use of skin biopsy in distinguishing IPD from MSA and other forms of parkinsonism. No attempt was made to correlate p-α-Syn deposition to disease severity or duration. Once again, a larger study would be of value in validating these results and developing wider datasets before its use can be supported in clinical practice. Mean disease duration varied considerably between clinical groups (MSA 4.9 years; IPD 12.4 years; tauopathies 3.6 years) and future studies may consider matching for both age and diseased duration.

Doppler MD et al. (2015) Mov Disord 30(12):1688–1692.

## Phosphorylated alpha-synuclein in skin nerve fibres differentiates Parkinson’s disease from multiple system atrophy

Zange et al. addressed the hypothesis that p-α-Syn can be detected in skin sympathetic nerve fibres in Parkinson’s disease, but not MSA. This study included 10 patients with IPD (according to the UK Parkinson’s Disease Society Brain Bank criteria) and 10 patients with MSA (according to Gilman criteria). Clinical classification was further supported by nuclear imaging and autonomic reflex screening. Six patients with an essential tremor served as controls.

Three-millimetre punch biopsies were taken from the forearm of the clinically more affected side. p-α-Syn deposits were detected in nerve fibres innervating skin autonomic structures in α-Syn-dependent neurodegenerative disorders and p-α-Syn deposition was limited to Parkinson’s disease. However, dermal nerve fibres of MSA and control patients were devoid of p-α-Syn. The authors conclude that as their study did not show involvement of postganglionic sympathetic skin nerve fibres in MSA (with a clinical duration up to 7 years), testing sympathetic skin nerve fibres for p-α-Syn deposits is of value in differentiating Parkinson’s disease from MSA. However, they suggest that further studies of a variety of disease durations are necessary to verify this. Their data support the finding of dermal denervation in Parkinson’s disease, occurring independently of age and correlating with the extent of intra-axonal p-α-Syn deposition.

*Comment.* This study supports the use of skin biopsy to differentiate IPD from MSA, specifically looking at autonomic skin nerve fibres. However, perhaps the most relevant conclusion that can be drawn is that autonomic skin fibre biopsies can be used to differentiate IPD from ‘not IPD’. The Parkinson’s disease patients in this study had a mean disease duration of 5.85 years, compared to 4.10 years for the MSA patients which are relatively well matched, although mean age of the cohorts differed by almost 10 years. As in the previous two papers, this study also supports the relationship of dermal denervation to p-α-Syn deposition. It would clearly be of value to obtain longer term follow-up data on these cohorts including pathology if available, to verify diagnosis and pathological conclusions.

Zange et al. (2015) Brain 138:2310–2321.

